# Deprescribing Strategies: A Prospective Study on Proton Pump Inhibitors

**DOI:** 10.3390/jcm12083029

**Published:** 2023-04-21

**Authors:** Giulia Calvini, Giammarco Baiardi, Francesca Mattioli, Giulia Milano, Francesca Calautti, Alessia Zunino, Carla Elda Fraguglia, Fabio Caccavale, Francesca Lantieri, Giancarlo Antonucci

**Affiliations:** 1 Clinical Pharmacology Unit, E.O. Ospedali Galliera, Mura Delle Cappuccine, 14, 16128 Genoa, Italy; 2 Clinical Pharmacology and Toxicology Unit, Department of Internal Medicine, University of Genoa, Viale Benedetto XV, 2, 16132 Genoa, Italy; 3 Department of Laboratory Medicine, IRCCS Ospedale Policlinico San Martino, 16132 Genoa, Italy; 4 S.C. Farmacia Interna, E.O. Ospedali Galliera, Mura Delle Cappuccine, 14, 16128 Genoa, Italy; 5 Chartered Accountancy and Advisory Firm, Piazza Remondini 3, 16131 Genoa, Italy; 6 Biostatistics Unit, Health Science Department, University of Genoa, Via Pastore 1, 16132 Genoa, Italy; 7 Internal Medicine Unit, E.O. Ospedali Galliera, Mura Delle Cappuccine, 14, 16128 Genoa, Italy

**Keywords:** polypharmacy, prescriptive appropriateness, deprescribing, proton pump inhibitors, clinical pharmacology

## Abstract

Proton pump inhibitors (PPIs) are among the most controversially prescribed drugs in polypharmacy. This observational prospective study assessed the PPI prescriptive trend during hospitalization before and after implementation of a prescribing/deprescribing algorithm in a real-life hospital setting and the related clinical–economic benefit at discharge. PPI prescriptive trends were compared between three quarters of 2019 (9 months) and the same period of 2018 by a chi-square test with a Yate’s correction. The proportions of treated patients in the two years (1120 discharged patients in 2018 and 1107 in 2019) were compared by the Cochran–Armitage trend test. DDDs (defined daily doses) were compared between 2018 and 2019 by the non-parametric Mann–Whitney test and normalizing DDD/DOT (days of therapy) and DDD/100 bd (bed days) for each patient. Multivariate logistic regression was performed on PPI prescriptions at discharge. The distribution of patients with PPIs at discharge was significantly different in the two years (*p* = 0.0121). There was a downward trend in the number of PPI prescriptions (29.9%) in the third trimester of 2019 compared to the others of the same year (first trimester: 34.1%, second trimester: 36.0%) and by contrast with the same periods of 2018 (29.4, 36.0, and 34.7%) (*p* = 0.0124). DDDs/patient did not differ between 2018 and 2019 nor across the three trimesters. However, both DDD/DOT and DDD/100 bd showed a decrease in the third trimester of 2019, with a marked difference for DDD/DOT (*p* = 0.0107). The reduction in consumption detected in the last phase of 2019 in terms of DDD/DOT was 0.09 with a consequent containment of pharmaceutical spending. The development and implementation of multidisciplinary prescribing/deprescribing protocols in both hospital and community settings could lead to a reduction in the misuse of PPIs, with significant savings in healthcare resources.

## 1. Introduction

Polypharmacy, understood as the prescription of more than five drugs to be taken daily by the same patient, is a constantly increasing phenomenon. The number of prescribed medicines is particularly high in the elderly population, as aging brings a number of chronic conditions that need specific treatment [1]. In industrialized countries, the risk of hyper-medicalization has become a challenging health problem. Although prescribing a treatment following the guidelines for a specific disease may be appropriate and have a favorable benefit/risk ratio, the prescribing physician should not forget to weigh the extent to which a new drug prescription may affect the whole therapy and, consequently, the general state of the patient. This is especially relevant in polypathological situations that can involve different guidelines; this aspect is also influenced by legal medicine that frequently forces prescribers to adhere to guidelines to protect themselves [2,3]. Nonadherence to therapy, medication errors, drug–drug interactions (DDIs), prescribing cascades [4,5], and increases in adverse drug reactions (ADRs) [6] have a negative impact on patients’ health and quality of life. Furthermore, this situation has a relevant impact on the costs that a healthcare system incurs to provide the medicinal products initially, and subsequently, paradoxically, to manage the ADRs and the consequences of therapy failure due to nonadherence [7].

The consumption of medicinal products in Italy over the last 17 years shows an upward trend, going from 763.8 DDDs (defined daily doses)/1000 inhabitants per day in 2004 to 993.1 DDDs/1000 inhabitants per day in 2020. A total of 98% of the elderly (over 65 years) receive at least 1 drug prescription during the year, and of these, 65.8% take at least 5 drugs and 26.1% are being treated with at least 10 different drugs [8,9]. The national usage trend, for all drugs treating peptic ulcers disease (PUD) and gastroesophageal reflux disease (GERD), grew constantly, going from 79.9 to 82.7 DDDs/1000 inhabitants per day during the 2017–2020 period. In the Italian general population, proton pump inhibitors (PPIs) are among the first 15 drugs in terms of consumption and among the first 10 drugs in terms of costs sustained by the Italian National Health System (NHS). In 2019, pantoprazole, lansoprazole, omeprazole, and esomeprazole accounted, all together, for 80.5 DDDs/1000 inhabitants per day [9,10]. The inappropriate use of PPIs contributes to generating polytherapies and a series of systematic reviews assert that PPIs are over-prescribed and misused. A possible explanation for this phenomenon could be the mistaken belief that these medicines have few side effects [11,12,13]. Unfortunately, and conversely, numerous studies have documented a close causal link between the use of PPIs and ADRs. Moreover, DDIs can also occur with the use of PPIs [14,15]. As these issues have become an important problem worldwide, both regulatory authorities and researchers are making efforts to promote adequate prescribing and even deprescribing. These two actions have the same aim of improving the clinical outcomes and treatment adherence of patients. As an attempt to address appropriate prescribing, the Italian Medicines Agency (AIFA) has released several “Notes”, which are regulatory tools to restrict the full reimbursement by the NHS of specific drug costs only when prescribed for the specific diseases approved in the indications. Therefore, if a medicine is prescribed for a disease not included in the list of AIFA Notes, its cost will not be reimbursed, and the patient will have to pay for the cost of the therapy out of pocket [16]. Two AIFA Notes (1,48) currently regulate the prescription of PPIs [17,18]. Deprescription strategies, in contrast, mainly entail reducing or interrupting drug administration by rationalizing polypharmacy therapies. Finally, an adequate monitoring period is desirable to verify the effectiveness of the implemented interventions [19]. To be successful, deprescribing strategies must be based on proactive interventions, which must be prolonged over time, structured, and supported by institutions, with the involvement not only of hospital medical structures but also of territorial medical structures, thus ensuring continuity of care. Moreover, these interventions have to include useful tools for making evidence-based decisions, shared by the entire care team, as well as by the patient. A deprescription flowchart could be a helpful tool to monitor prescriptions and create guidelines that are widely applicable. Barbara Farrell and colleagues from the University of Ottawa have created a flowchart aimed at reducing PPI prescriptions, in line with current treatment indications, which offers doctors recommendations and clinical suggestions to suspend/reduce the prescription of PPIs [20].

The primary aim of the study was the assessment of the prescriptive trend of PPIs during hospitalization before and after the introduction of the Farrell et al.’s flowchart to a real-life hospital setting in the Department of Internal Medicine of E.O. Ospedali Galliera (Genoa, Italy). The secondary objective was to evaluate this strategy influence on PPIs trend of prescriptions at hospital discharge and the related economic impact.

## 2. Materials and Methods

This observational prospective study, approved by the Regional Ethics Committee (authorization nr. 032/2019), did not provide for the execution of any invasive method or variation of the normal ward clinical practice. The pattern of PPI prescriptions was analyzed before, during, and after the application of the prescription/deprescription flowchart created by B. Farrell and colleagues [20], validated in Italian and slightly modified to comply with AIFA Notes 1 and 48 [17,18]. Note 1 provides for the Italian NHS reimbursability of PPIs for the prevention of serious upper gastrointestinal tract complications in patients chronically treated with nonsteroidal anti-inflammatory drugs (NSAIDs) or on antiplatelet therapy with low-dose acetylsalicylic acid, provided at least one risk condition is met (previous digestive hemorrhage or PUD not healed with eradicant therapy, concomitant anticoagulant/cortison therapy, advanced age). Note 48 provides for the reimbursability of PPIs only for specific clinical conditions (e.g., first episode of GERD with or without oesophagitis; Zollinger–Ellison syndrome) and establishes the maximum duration of treatment.

This study was divided into three phases of a duration of three months each in order to evaluate the prescriptive trend of PPIs, without a seasonal bias of PUD flares, since gastrointestinal (GI) diseases’ seasonality has been observed [21,22,23]. The consumption of PPIs was evaluated in and between quarters of 2019 for a total of 9 months (January–March (Phase 1), April–June (Phase 2) and September-November (Phase 3)). Then, a further analysis of PPIs prescription trend was made with the same quarters of the previous year (2018) as a comparison. The periods of July–August and December were excluded to avoid periods where holidays and different customs might affect normal hospitalization and prescription trends. 

During the first phase (Phase 1, training–preliminary data collection), the study design was explained and discussed with the multidisciplinary team, consisting of clinical pharmacologists, internists, nurses and pharmacists; meetings were held to raise awareness of the problem of polytherapy and the possibility of DDIs and thus the need to rationalize prescriptions. Then, data on PPI prescriptions were collected prior to the delivery of the flowchart (from 1 January 2019 to 31 March 2019). 

During Phase 2 (active study phase–implementation of the flowchart), from 1 April 2019 to 30 June 2019, the flowchart application was implemented, with the presence of clinical pharmacologist in the Internal Medicine wards. The registered patients were prescribed/deprescribed a PPI according to clinical needs and physician evaluation. In any case, the flowchart represented a mere therapeutic suggestion that could be accepted or ignored by the care provider. In this way, the patient was always guaranteed the best therapy in relation to clinical symptoms/needs, based on the sensitivity and readiness of the caregiver. 

In Phase 3 (post-intervention observational phase–final analysis of the results), from 30 September 2019 to 30 November 2019, the deprescription flowchart was applied to all inpatients with a PPI prescription as routine clinical practice. 

Age, sex, length of hospital stay, PPIs administered (active ingredient, dosage, period of administration, number of tablets administered to each patient), and number of patients with PPIs in discharge therapy were evaluated in all phases. 

Pantoprazole (PAN) and lansoprazole (LAN) are the PPIs available to the attending physician in Galliera Hospital. PAN is marketed in Italy in oral formulations of 20 mg (PAN20) and 40 mg (PAN40); LAN is marketed in 15 mg (LAN15) and 30 mg (LAN30) oral formulations. PAN40 for intravenous use, which is provided by the hospital pharmacy and is available to clinicians, is used in situations of particular severity and acuity; therefore, PPI prescriptions for intravenous use were not considered in this analysis. Since it is common practice to transfer patients between wards based on the diagnosis, the clinical course, and the needs of the hospital, we considered in the analysis the discharge from the ward and not the admissions. Moreover, the percentage (%) of patients discharged with a PPI was calculated by excluding all those subjects who died during hospitalization, despite having taken PPIs.

PPI consumption data were extracted from the hospital administrative database and are expressed in terms of DDD (defined daily dose). Italian national and regional consumption, expenditure, and average cost data were collected through the so-called OsMed-AIFA information flow [9,10]. In order to quantify the reduction or overspending resulting from the consumption of PPIs, the following indicators for economic evaluation were considered: DDD/patient, expressing a patient’s PPI treatment exposure according to hospitalization period, and DDDs divided by days of PPI therapy (DDD/DOT) and by hospitalization days (DDD/100 bd), expressed as DDDs per 100 bed days (“a day during which a person is confined to a bed and in which the patient stays overnight in a hospital”, as defined by the World Health Organization), counting the days of admission and discharge as two bed days [8,24].

We calculated the indicator DDD/DOT using the following formula:

$$\frac{\sum \mathrm{DDD}}{\sum \mathrm{days}\;\mathrm{of}\;\mathrm{treatment}}$$

and calculated the indicator DDD/100 bd (%) using the following formula:

$$\frac{\sum \mathrm{DDD}}{\sum \mathrm{bed}\;\mathrm{days}}\times 100$$

### Statistics

Continuous variables are reported as mean and standard deviations (SDs), while categorical data are reported as counts and percentages. The total number of patients hospitalized, the number of patients treated with PPI during hospitalization, and the number of patients discharged with PPI indications were compared between 2018 and 2019 for the whole nine-month period by a chi-square test with a Yate’s correction. The percentages of male and female patients discharged with PPIs in the two years for the whole year period and for each trimester were also compared by a chi-square test with a Yate’s correction. The proportions of patients treated with PPIs across the three trimesters, calculated with respect to the total number of inpatients for the same period, were compared in the two years by the Cochran–Armitage trend test. The same was performed for patients with PPI prescriptions at the time of hospital discharge, with drug withdrawal periods (defined as no PPI administration for at least one day during the therapy period), or by contrast, with continuous therapy, as well as for patients who received double doses (defined as those who received at least three drug administrations more than the number of days of therapy, no matter what dosage formulation or what specific PPI drug).

DDD indicators were compared between 2018 and 2019 for each trimester by the nonparametric Mann–Whitney test, because of the non-normal distribution of data. We carried out the comparison also normalizing DDD/DOT and DDD/100 bd for each patient.

Finally, multivariate logistic regression was performed on PPI prescriptions at the time of hospital discharge including patients’ age and gender, as well as the year and the trimester of discharge from hospital in the model as predictors. The analysis was repeated with data for 2018 and 2019 separately. Statistical significance was set at α = 0.05. Tests are two-tailed. Nominal *p*-values are reported.

## 3. Results

Regardless of PPI treatment, 1120 patients were discharged from the Internal Medicine ward in the 9 months of 2018 and 1107 in the 9 months of 2019, respectively. The data of the patients treated with PPI and discharged with a PPI prescription in the two years under comparison, evaluated for each quarter, are summarized in Table 1.

The percentages of use of the two active ingredients (PAN and LAN) considered in this study, at the different doses used in the two reference years, are shown in Table 2. The percentages refer to a total of 942 and 881 patients in 2018 and 2019, respectively, higher than the number of patients treated with PPIs in the two years, because 34 patients (19 in 2018 and 15 in 2019) received PPIs at 2 different dosages and were thus considered for both dosages.

Given that PAN40 is the PPI predominately utilized (77.6% and 79.6% in 2018 and 2019, respectively), it was decided not to develop the analysis by subdividing patients according to the molecule used, as any result would be scarcely comparable and lack statistical meaning.

In 2018, 923 patients were being treated with PPIs during their hospitalization (out of the 1120 patients discharged in the 9 months of 2018). In 2019, significantly fewer patients were treated with PPIs during their hospitalization (866 patients) vis-à-vis all the patients discharged in the 9 months that were studied (1107 patients), compared to 2018 (78.2% vs. 82.4%, *p* = 0.0152).

**Figure 1:**
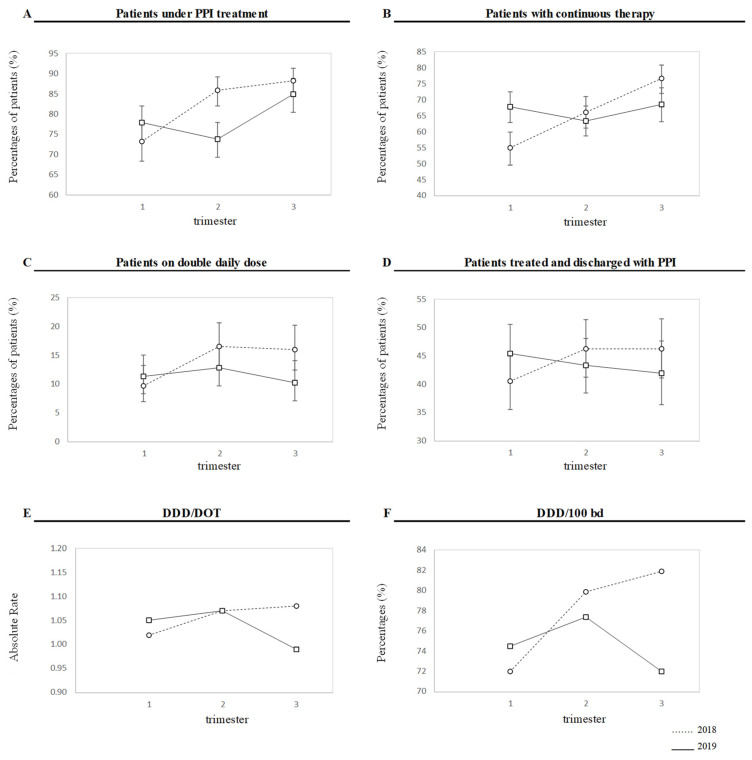
Percentages of patients in each trimester were calculated with respect to the total number of inpatients for each trimester/year. Panel (**A**), patients under PPI treatment; panel (**B**), patients with continuous therapy; panel (**C**), patients on double daily dose; panel (**D**), patients treated and discharged with PPI. 95% CI bars are also shown. DDD/DOT and DDD/100 bd (panel (**E**,**F**)) are shown as absolute rates and percentages, respectively (see text for details). Data for 2018 are shown with a dashed line, while data for 2019 are shown with a solid line.

Analyzing the data by trimester and year, it is clear that during the intervention period when the clinical pharmacologist was present in the ward (Phase 2; April–June, 2019), the percentage of patients in PPI therapy decreased (73.8%) compared to the other trimesters of the same year and the same trimester (April–June) of 2018 (see Table 1 and Figure 1 for details).

The data distribution analysis across the three trimesters comparing the two years is summarized in Table 3: in Phase 3 of 2019 (September–November, 2019), there was a downward trend in the number of patients with a PPI prescription (29.9%) compared to the other trimesters of the same year (34.1% and 36.0% for the first two trimesters), by contrast with the same period of 2018 (29.4, 36.0, and 34.7%) (*p* = 0.0124; Table 3). The same approach used for patients treated with PPI was applied to patients with continuous periods of therapy, with double daily doses and PPI prescriptions at the time of hospital discharge. In detail, the distribution of the number of patients taking PPIs in continuous therapy between trimesters (737 Pts in 2018 vs. 734 Pts in 2019) has significantly different proportions in the three trimesters of 2018 and 2019, with a decrease in trimester 3 of 2019 (*p* = 6 × 10^−5^, Table 3).

Analysis of the data shows that in Phase 3 of 2019 there was also a sharp drop in double-dose PPI administration with an absolute value (n.31) that is the lowest recorded in the periods studied and that was not observable in the previous year (*p* = 0.0106; Table 3).

The number of patients for whom the drug was confirmed at the time of hospital discharge compared to all patients with a PPI prescription, net of deceased patients, is not statistically different in the two years of observation for none of the trimesters, although a downward trend is evident between trimesters and for each year. In any case, in Phase 3 of 2019, the lowest percentage (53.1%) of patients with PPIs at the time of hospital discharge was recorded (128 Pts) compared to patients being treated during their hospitalization, net of deceased patients (241 Pts, 18 Pts deceased) (Table 1).

Statistical analysis demonstrates that the distribution of patients with PPIs at the time of hospital discharge between trimesters is significantly different in the two years (*p* = 0.0121; Table 3). Moreover, it is interesting to note that the percentage of patients discharged with PPIs in Phase 3 of 2019, after the systematic application of the abovementioned deprescription protocol, was the lowest recorded (26.5%; 128 Pts out of 483 Pts). To note, although proportions of patients under PPI treatment comparing the three trimesters of each year showed a decrease in Phase 3 of 2019, the proportions in each trimester with respect to the total number of patients hospitalized showed instead an increase in the third trimester (84.9% for the third quarter vs. 73.9% for the second quarter of 2019). However, this was partly due to a seasonal effect, as it was observable also in 2018. In addition, these patients included those already under PPIs at hospital admission. Looking at the percentages of patients dismissed from hospital with PPIs, which better reflects the flowchart implementation effect, there was a decrease during Phase 2 and Phase 3 in 2019 (from 45.4% to 43.3% and 42.0%, respectively) not observable in 2018, the year of control (from 40.5% to 46.3% and 46.3%, as expected based on the seasonal fluctuation). We similarly observed a decrease in the percentages with respect to the total number of hospitalized patients compared to 2018 in patients with a double daily PPI dose and in DDT/DOT and DDD/100 bd (Figure 1). These trends were even more marked when referred to patients under PPI treatment instead of total hospitalized patients (Table 1).

The percentages of females and males with PPI prescriptions at the time of hospital discharge was highly variable across the three trimesters; however, females were generally discharged with PPI prescriptions less often than males in 2018 (54.1% of females vs. 63.0% of males, *p* = 0.0110), variably but still consistently across the three periods, while in 2019 this difference faded away (59.5 vs. 60.3%).

DDDs/patient did not differ between 2018 and 2019, nor across the three trimesters (Table 1). However, both DDD/100 bd and DDD/DOT showed a decrease in Phase 3 of 2019, which was not observable at all in 2018 (Table 1). DDD/100 bd declined in the September–November period from 81.9 in 2018 to 72.0 in 2019. DDD/DOT in Phase 3 of 2019 was lower (0.99) than that for the same period of the previous year (1.08) and this reduction (amounting to 0.09) is statistically significant (Table 1, Figure 1). As a matter of fact, also calculating the DDD/DOT and DDD/100 bd for each patient, we observed an apparent decrease after the second semester in 2019 but not in 2018 (mean ± standard deviation: 1.00 ± 0.36, 1.03 ± 0.38, and 1.06 ± 0.40 for the three trimesters, respectively, in 2018 vs. 1.02 ± 0.34, 1.03 ± 0.38, and 0.98 ± 0.32 in 2019 for both DDD/DOT and 0.73 ± 0.40, 0.78 ± 0.37, and 0.81 ± 0.44 in 2018 vs. 0.76 ± 0.39, 0.77 ± 0.40, and 0.73 ± 0.36 in 2019 for DDD/100 bd), and a marked difference between the two years only for the third trimester, which was statistically significant for DDD/DOT (1.06 vs. 0.98, *p* = 0.0107).

To further evaluate the change in PPI prescription at the time of hospital discharge in relation to the flowchart application in Phase 2, we performed a multivariate logistic regression, adjusting for gender and age. Analyzing 2018 and 2019 data together, logistic regression indicated that there was a significant association between PPI prescription at discharge and age (the higher the age the higher the PPI prescription probability, *p* = 0.0202), gender (females were less likely to be prescribed with PPI at discharge, *p* = 0.0306), and trimester of discharge from hospital (trimesters later in the year had a lower probability of PPI prescription, *p* = 0.0067), while the year of hospitalization did not contribute at all to the model (Table 4). Analyzing the two years separately, for 2018 neither the age nor the trimester of discharge were significantly related to PPI prescription, with only gender still significant—OR (95%CI): 0.68 (0.52–0.90); *p* = 0.0069. By contrast, for 2019 gender was no longer significant, while age and the trimester were weak yet significant predictors: OR (95% CI) of 1.01 (1.00–1.03) and *p* = 0.0186 for age and OR (95% CI) of 0.82 (0.68–0.98) and *p* = 0.0257 for the trimester (Table 4).

## 4. Discussion

Specific prescription rationalization programs can prove to be a useful and effective tool for reducing the consumption of drugs that are widely used, often without being indicated.

Examining our data, patients treated with PPIs during each of the 9 months studied accounted for 82.4% of patients in 2018 and a lower proportion of 78.2% in 2019. During the second trimester of 2019 (Phase 2), we noted that the percentage of patients in therapy with PPIs (73.8%) was the lowest compared with the other trimesters of the same year and the corresponding trimester of 2018. It is also worth noting that, in an analysis of the distribution of patients in therapy in the various trimesters for each year, the lowest percentage of patients in therapy with PPIs and discharged from hospital with a PPI prescription was recorded in the third trimester of 2019 (Phase 3, 53.1% Table 1). In our opinion, it supports the thesis, according to which multidisciplinarity is the keystone for good management of therapies, in particular in the current health context in which there is an increasingly higher number of available drugs and a growing number of polypathological and polytreated patients. Other interesting data relate to the net reduction in double-dose intake in the third trimester of 2019. Despite PPIs having a relatively short half-life, which is about 2 h for all molecules, they irreversibly bind to their targets, forcing the gastric parietal cell to synthesize anew the proton pumps. Their effect is therefore prolonged and exceeds 24 h. Thus, daily double-dose administration is unjustified, apart from in exceptional cases.

Indicators based on the number of DDDs consumed are useful to quantify the intensity of the use of a drug or group of drugs in a given context of analysis, once consumption or prescriptions have been detected. The relationship between the number of DDDs consumed by each patient and treatment days (DDD/DOT) in relation to the consumption of PPI in Phase 3 of 2019 was lower than that for the same period of the previous year: indeed, the value went from 1.08 to 0.99 and this reduction (amounting to 0.09) is statistically significant. Similar differences can be seen in the indicator that parametrizes the intake of PPI with hospitalization days in the same unit of time: the percentage of patients treated with PPI, that is, the number of daily treatments administered every 100 days of hospitalization (DDD/100 bd), declined in the September–November period from 81.9 in 2018 to 72.0 in 2019. Modifying therapeutic orientation and prescription habits led to noticeable changes in the intensity of PPI use: the reduction in consumption detected in the last phase of 2019 in terms of DDD/DOT was 0.09 with a consequent containment of pharmaceutical spending, at least in relation to patients treated and discharged from the ward in Phase 3 of the study.

Finally, since the study deals with hospitalized patients, it was important to analyze the percentage of patients discharged with PPI prescriptions. Although the percentage of patients discharged with PPI vis-à-vis the total number of patients treated with PPIs differed in the two considered years (53.1% discharged patients with PPI out of the total treated in 2019 compared to 55.3% in 2018), it was not statistically significant and therefore irrelevant in terms of the immediate impact on territorial healthcare. However, the analysis of the distribution seems to confirm the utility of the intervention carried out, considering the lower percentage of patients treated and discharged with PPIs in the post-intervention trimester (26.5%). The multiple logistic regression showed that in 2019 age and the trimester of discharge were significant predictors for PPI prescription at the time of discharge: younger patients and patients discharged in trimesters later in the year, which correspond to Phase 2 and Phase 3, had a lower probability of PPI prescriptions. By contrast, in 2018, when the flowchart had not yet been applied, only the patients’ gender was significant, with male patients receiving a prescription more often than females (63.0 vs. 54.1%), as also highlighted by the logistic regression, while PPI prescription was not significantly related to the age or to the trimester of discharge.

It is reasonable to hypothesize that the greatest impact in both health and economic terms can be seen in the chronic and continued use (years/decades) of PPIs in territorial healthcare. It is worth noting that hospital applications of deprescription protocols, however important, cannot replace the far more difficult task of transferring a more judicious prescriptive thought model to territorial healthcare. Naturally, hospitals are very different to territorial healthcare, not only because all of the prescribed drugs are actually administered, but also because physicians work in a smaller, controlled environment. In particular, the clinical conditions of hospitalized patients are certainly more severe, and the size of the hospital population is much smaller than that of a whole area of interest of territorial healthcare.

Consumption data expressed in DDDs/1000 inhabitants/day provide an estimate of the proportion of the population in a given geographical area exposed daily to a specific drug. In the case of drugs like PPIs, which, despite having a limited number of indications and a limited dose range, are often used improperly for extended periods, the value of the theoretical exposition of the population is very close to the real one and takes on considerable epidemiological significance. The total expenditure of the Italian NHS on PPIs in 2020 was EUR 661.40 million and PPI consumption in terms of DDDs/1000 population/day was 70.80 [9]; thus, in a population of about 59.5 million inhabitants, the DDD usage of PPIs in 2020 totaled EUR 1,538,140,000 with an average DDD cost of EUR 0.43. With a hypothetical reduction of 0.09 in unit DDD consumption as shown in Phase 3 of the study, due to interventions aimed at promoting an appropriate use of drugs, it is possible to estimate national cost savings of about EUR 841,000 per annum, at an unchanged cost per DDD. As well as the health gains, a reduction in PPI consumption would also enable a significant saving of resources, which could be used in other contexts, especially in regions affected by difficult financial circumstances and subject to health deficit recovery plans.

The analysis of our data demonstrates that overall, both for 2018 and 2019, about 80% of inpatients were prescribed a PPI during their hospitalization, and 60% of these had it confirmed at the time of discharge. Since this study is based on real-life data, several sources of variability might have affected the results. Lengths of hospitalization and of PPI treatment were highly variable, possibly overlapping different months and even different trimesters. Our data are aggregated on a trimester basis to reduce such a fluctuation while still taking into account possible seasonal effects; nonetheless, the resolution related to a shorter period definition is lost. In addition, since the aggregated data refer to a two-year-long period, we cannot exclude a secular trend in drug prescriptions that might have affected the rate of patients’ admission and PPI prescription. Finally, the particular frailty conditions of inpatients compared with those of patients followed in the territory might have influenced the clinician’s different approach to the cure. In addition to the above limits, the reduction in hospital use of PPIs certainly does not represent the cornerstone of our intervention, but greater prescriptive attention can play a fundamental role in transferring the right message from the hospital to territorial healthcare. That PPI therapy was not confirmed at the time of discharge for about 40% of patients leads us to conclude that, rather than a real necessity for gastric protection, a preventative/defensive attitude with scant or no scientific basis lies at the basis of intra-hospital prescription. A possible explanation for this data is the commonly held idea that it is necessary to associate a PPI with drugs such as antiaggregants, cortisone, or antibiotics, which are often used during hospitalization [25]. It seems superfluous to point out that administering a drug that is not indicated exposes patients to a greater risk of side effects and interactions among drugs without bringing any additional clinical benefit [26,27].

## 5. Conclusions

The excessive and unnecessary use of PPIs, as well as its high impact in terms of adherence, patient quality of life, and expenditure for the National Health System, requires advanced strategies aimed at identifying the best clinical practices to be implemented in different care settings. The development and implementation of multidisciplinary prescribing/deprescribing protocols in both hospital and community settings could lead to an improvement in the appropriateness of PPI use, with significant savings in healthcare resources.

## Figures and Tables

**Table 1:** Data of Patients treated with PPIs.

**Year 2018**	**9 months**			**January–March**					**April–June**					**September–November**				
**Patients TOTAL (*n*)**	**1120**			**370**					**387**					**363**				
**Patients in PPI (*n*, %)**	923		**82.4**	271			**73.2**		332				**85.8**	320			**88.2**	
**Age (mean ± SD)**	76.5	±	12.1	78.2	±		10.3		75.6	±			12.9	75.9	±		12.5	
**Females (%)**	47.3			52.0					43.7					47.2				
**Total days of hospitalization (mean ± SD)**	15.7	±	11.3	15.9	±		11.0		16.8	±			13.6	14.4	±		8.7	
**Pts deceased during hospitalization (*n*)**	78			32					30					16				
**Days of PPI administration (mean ± SD)**	11.6	±	9.5	11.2	±		9.0		12.5	±			11.2	10.9	±		7.9	
**Pts with continuous therapy (*n*, %)**	737		79.8	203			74.9		256				77.1	278			86.9	
**Pts on double daily dose [>3 gg] (*n*, %)**	158		**17.1**	36			**13.3**		64				**19.3**	58			**18.1**	
**DDD/patient (mean ± SD)**	12.3	±	12.7	11.4	±		11.1		13.4	±			15.6	11.8	±		10.3	
**DDD/DOT (*n*)**	**1.06**			**1.02**					**1.07**					**1.08**				
**DDD/100bd (%)**	**78.2**			**72.0**					**79.9**					**81.9**				
Pts treated and discharge with PPI (*n*, % *)	497		**58.8**	150			**62.8**		179				**59.3**	168			**55.3**	
**Pts discharge with PPI *versus* total Pts. discharged (%)**			**44.4**				**40.5**						**46.3**				**46.3**	
**Year 2019**	**9 months**			**January–March**					**April–June**					**September–November**				
**Patients TOTAL (*n*)**	**1107**			**379**					**423**					**305**				
**Patients in PPI (*n*, %)**	866		**78.2**	295				**77.8**	312			73.8		259				**84.9**
**Age (mean ± SD)**	75.7	±	12.3	75.2		±		14.3	76.7		±	10.4		75.0		±		12.0
**Females (%)**	42.8			41.7					39.1					48.6				
**Total days of hospitalization (mean ± SD)**	16.2	±	12.4	15.0		±		10.4	16.9		±	13.5		16.6		±		13.2
**Pts deceased during hospitalization (*n*)**	61			23					20					18				
**Days of PPI administration (mean ± SD)**	11.6	±	9.5	10.6		±		7.8	12.3		±	10.7		12.1		±		9.6
**Pts with continuous therapy (*n*, %)**	734		84.8	257				87.1	268			85.9		209				80.7
**Pts on double daily dose [>3 gg] (*n*, %)**	128		**14.8**	43				**14.6**	54			**17.3**		31				**12.0**
**DDD/patient (mean ± SD)**	12.1	±	12.0	11.2		±		10.4	13.1		±	14.0		12.0		±		11.1
**DDD/DOT (*n*)**	**1.04**			**1.05**					**1.07**					**0.99**				
**DDD/100bd (%)**	**74.8**			**74.5**					**77.4**					**72.0**				
Pts treated and discharge with PPI (*n*, %*)	483		**60.0**	172				**63.2**	183			**62.7**		128				**53.1**
**Pts discharge with PPI *versus* total Pts. discharged (%)**			**43.6**					**45.4**				**43.3**						**42.0**

Notes. Pts on double daily dose (>3 gg), patients received at least three drug administrations more than the number of days of therapy; *, % calculated excluding patients who died during hospitalization. Abbreviations: Pts, patients; *n*, absolute numbers; %, percentage; SD, standard deviation; DDD/patient, DDD/DOT, DDD/100 bd, see text for definition.

**Table 2:** Data of Patients treated with PPIs.

Year 2018—9 months																				
[Patients TOTAL (*n*): 1120]	**PPI (Total)**			**LAN15**				**LAN30**				**PAN20**				**PAN40**				
**Patients in PPI (*n*, %)**	**923**		**82.4**		**2**		**0.2**		**84**		**9.1**		**140**		**15.2**		**716**		**77.6**	
**Age (mean ± SD)**	76.5	±	12.1		88.0	±	2.8		79.2	±	10.2		79.0	±	11.4		75.6	±	12.3	
**Females (%)**	47.3				0				44.0				51.4				47.1			
**Year 2019—9 months**																				
[Patients TOTAL (*n*): 1107]	**PPI (Total)**			**LAN15**				**LAN30**				**PAN20**				**PAN40**				
**Patients in PPI (*n*, %)**	**866**		**78.2**		**2**		**0.2**		**48**		**5.5**		**142**		**16.4**		**689**		**79.6**	
**Age (mean ± SD)**	75.7	±	12.5		90.0	±	4.2		15.5	±	12.7		76.9	±	12.8		75.2	±	12.6	
**Females (%)**	42.5				100.0				45.8				40.8				43.0			

Notes. Pantoprazole (PAN) and lansoprazole (LAN) are used in the clinical routine at the Galliera Hospital; PAN is marketed in Italy in oral formulations of 20 mg (PAN20) and 40 mg (PAN40); LAN is marketed in two oral formulations of 15 mg (LAN15) and 30 mg (LAN30). Abbreviations: *n*, absolute numbers; %, percentage; SD, standard deviation.

**Table 3:** Percentages (95% CI) of patients with respect to the total number of inpatients for each trimester/year.

		N				% (95%CI)			
	yr.	Tot.	Phase I	Phase II	Phase III	Phase I	Phase II	Phase III	*p*-Value *
**Patients under PPI** **treatment**	**2018**	923	271	332	320	73.2 (68.4–77.7)	85.8 (81.9–89.1)	88.2 (84.4–91.3)	0.0124
	**2019**	866	295	312	259	77.8 (73.3–81.9)	73.8 (69.3–77.9)	84.9 (80.4–88.7)	
**Patients with continuous therapy**	**2018**	737	203	256	278	54.9 (49.6–60.0)	66.1 (61.2–70.9)	76.6 (71.9–80.9)	<0.0001
	**2019**	734	257	268	209	67.8 (62.9–72.5)	63.4 (58.6–68.0)	68.5 (63.0–73.7)	
**Patients on double daily dose**	**2018**	158	36	64	58	9.7 (6.9–13.2)	16.5 (13.0–20.6)	16.0 (12.4–20.2)	0.0106
	**2019**	128	43	54	31	11.3 (8.3–15.0)	12.8 (9.7–16.3)	10.2 (7.0–14.1)	
**Patients treated and** **discharged with PPI**	**2018**	497	150	179	168	40.5 (35.5–45.7)	46.3 (41.2–51.4)	46.3 (41.1–51.6)	0.0121
	**2019**	483	172	183	128	45.4 (40.3–50.6)	43.3 (38.5–48.1)	42.0 (36.4–47.7)	

* *p*-values are calculated with the Cochran–Armitage trend test applied to the distribution across the three trimesters comparing the two years.

**Table 4:** Logistic regression of PPI prescription as predicted by gender, age, trimester, and year of data collection for 3 models: (i) with 2018 and 2019 together (summary), (ii) 2018 data, and (iii) 2019 data.

	Summary					2018					2019				
Predictor	B (S.E.)	Wald’s Chisquare (df)	*p*	OR	95%CI OR	B (S.E.)	Wald’s Chisquare (df)	*p*	OR	95%CI OR	B (S.E.)	Wald’s Chisquare (df)	*p*	OR	95%CI OR
**gender** **(ref = females)**	−0.216 (0.102)	4.52 (1)	0.0336	0.81	(0.66–0.98)	−0.382 (0.141)	7.29 (1)	0.0069	0.68	(0.52–0.90)	−0.034 (0.148)	0.05 (1)	0.8178	0.97	(0.72–1.29)
**age**	0.010 (0.004)	5.37 (1)	0.0205	1.01	(1.00–1.02)	0.005 (0.006)	0.80 (1)	0.3700	1.01	(0.99–1.02)	0.013 (0.006)	5.12 (1)	0.0237	1.01	(1.00–.03)
**year**	0.027 (0.101)	0.07 (1)	0.7861	1.03	(0.84–1.25)										
**trimester (ref = 1)**		7.99 (2)	0.0184				3.11 (2)	0.2111				6.10 (2)	0.0474		
**trimester 2**	−0.088 (0.125)	0.50 (1)	0.4786	0.92	(0.72–1.17)	−0.156 (0.180)	0.76 (1)	0.3846	0.86	(0.60–1.22)	−0.047 (0.175)	0.07 (1)	0.7887	0.95	(0.67–1.35)
**trimester 3**	−0.340 (0.126)	7.23 (1)	0.0072	0.71	(0.57–0.91)	−0.312 (0.178)	3.08 (1)	0.0791	0.73	(0.52–1.04)	−0.410 (0.182)	5.11 (1)	0.0238	0.66	(0.46–0.95)
**constant**	−0.326 (0.341)	0.92 (1)	0.3387	0.72		−0.070 (0.482)	0.02 (1)	0.8848	0.93		−0.470 (0.472)	0.99 (1)	0.3200	0.63	

The overall *p*-value for the logistic regression models is significant: *p* = 0.0026 for summary, *p* = 0.0268 for 2018, and *p* = 0.0182 for 2019 data. S.E. is the standard error of the beta regression coefficient (B). The odds ratio (OR) is calculated as the exponential of the B coefficient. The categorical reference (ref) is females for the gender and trimester 1 for the trimesters. Significant *p*-values (≤0.05) are in italics.

## Data Availability

The clinical and laboratory data supporting this study’s findings are available from the corresponding author F.M. upon special request. The datasets generated or analyzed during the current study are not publicly available for ethical reasons per local guidelines.
